# Correction: Universal control of proton concentration using an electrochemically generated acid compatible with miniaturization

**DOI:** 10.1039/d4na90034k

**Published:** 2024-03-25

**Authors:** Janwa El-Maiss, Divya Balakrishnan, César Pascual García

**Affiliations:** a Nanomaterials Unit of the Materials Research and Technology Department, Luxembourg Institute of Science and Technology (LIST) Belvaux L-4422 Luxembourg cesar.pascual@list.lu

## Abstract

Correction for ‘Universal control of proton concentration using an electrochemically generated acid compatible with miniaturization’ by Janwa El-Maiss *et al.*, *Nanoscale Adv.*, 2022, **4**, 3233–3242, https://doi.org/10.1039/D2NA00275B.

The authors regret the omission of acknowledgement of funders in the original manuscript. The following funders are acknowledged for financing the research for this manuscript: FNR ATTRACT project 5718158 NANOpH, and from the program H2020 Future Emerging Technologies of the European Commission under the grant No 862539 – Electromed-FET-Open.

The Royal Society of Chemistry apologises for these errors and any consequent inconvenience to authors and readers.

## Supplementary Material

